# Idiopathic Pulmonary Fibrosis: Aging, Mitochondrial Dysfunction, and Cellular Bioenergetics

**DOI:** 10.3389/fmed.2018.00010

**Published:** 2018-02-05

**Authors:** Daniel C. Zank, Marta Bueno, Ana L. Mora, Mauricio Rojas

**Affiliations:** ^1^Division of Pulmonary, Allergy and Critical Care Medicine, University of Pittsburgh School of Medicine, Pittsburgh, PA, United States; ^2^Vascular Medicine Institute, University of Pittsburgh School of Medicine, Pittsburgh, PA, United States; ^3^McGowan Institute of Regenerative Medicine, University of Pittsburgh School of Medicine, Pittsburgh, PA, United States; ^4^Dorothy P. & Richard P. Simmons Center for Interstitial Lung Disease, University of Pittsburgh School of Medicine, Pittsburgh, PA, United States

**Keywords:** aging, mitochondrial dysfunction, lung fibrosis, bioenergetics, mitophagy, senescence

## Abstract

At present, the etiology of idiopathic pulmonary fibrosis (IPF) remains elusive. Over the past two decades, however, researchers have identified and described the underlying processes that result in metabolic dysregulation, metabolic reprogramming, and mitochondrial dysfunction observed in the cells of IPF lungs. Metabolic changes and mitochondrial dysfunction in IPF include decreased efficiency of electron transport chain function with increasing production of reactive oxygen species, decreased mitochondrial biogenesis, and impaired mitochondrial macroautophagy, a key pathway for the removal of dysfunctional mitochondria. Metabolic changes in IPF have potential impact on lung cell function, differentiation, and activation of fibrotic responses. These alterations result in activation of TGF-β and predispose to the development of pulmonary fibrosis. IPF is a disease of the aged, and many of these same bioenergetic changes are present to a lesser extent with normal aging, raising the possibility that these anticipated alterations in metabolic processes play important roles in creating susceptibility to the development of IPF. This review explores what is known regarding the cellular metabolic and mitochondrial changes that are found in IPF, and examines this body of literature to identify future research direction and potential points of intervention in the pathogenesis of IPF.

## Introduction

Aging is life’s natural destiny. It is biologically defined by a progressive impairment in vital functions coupled with diminished fitness to adapt to environmental stimuli and respond to stress (homeostenosis) (1). Among the predictable cellular alterations that underlie homeostenosis, metabolic dysregulation and alterations in mitochondrial function have been shown to contribute to the aging phenotype. Aging is also associated with increased susceptibility to a wide range of chronic diseases. Lung pathologies are no exception, and the prevalence of several interstitial lung diseases, most notably idiopathic pulmonary fibrosis (IPF), has been found to increase considerably with age. IPF is a progressive and irreversible lung disease, generally diagnosed in the sixth decade of life, whose etiology remains unknown and therapeutic options remain limited (2). Many of the changes in bioenergetics and mitochondrial function seen with aging are also seen in the fibrotic lung and may contribute to IPF (Table 1).

**Table 1:** Mitochondrial changes in the fibrotic lung.

Feature	Change	Model	Reference
Mitochondrial reactive oxygen species	Increased	Bleomycin mouse model	(44)
		Asbestosis mouse model	

Mitochondrial respiration	Decreased ETC complex activity, lower OCR	Human idiopathic pulmonary fibrosis (IPF) lung tissue	(5, 15, 42, 43, 63, 66, 69)
		Human lung fibroblasts	
		Human AECII	
		Alveolar macrophages	
		MHV68 model of lung fibrosis	

ATP production	Decreased	IPF lung fibroblasts	(24, 43, 69)
		IPF lung myofibroblasts	
		IPF total lung	

mtDNA	Increased oxidative damage, insufficient mtDNA repair	IPF total lung	(5, 44, 48)
		Murine AECII	
		Bleomycin mouse model	
		Asbestosis mouse model	

Mitochondrial biogenesis	Decrease	IPF total lung	(15)
		Bleomycin mouse model	
Mitochondrial dynamics	Imbalanced	MHV68 mouse model of fibrosis	(5)

Mitophagy alterations	Reduced levels of mediators of mitochondrial quality control in epithelial cells and fibroblasts	IPF AECII IPF lung fibroblasts IPF pulmonary macrophages Bleomycin mouse model	(5, 24, 25, 30)
	Increased mitophagy in macrophages		
		MHV68 mouse model of fibrosis	

Decreased expression of SIRT3	Acetylation of mitochondrial proteins	IPF total lung	(48, 52)
		Bleomycin mouse model	
		Asbestosis mouse model	

*ETC, electron transport chain; OXPHOS, oxidative phosphorylation; AECII, alveolar type II epithelial cells; OCR, oxygen consumption rate*.

## Mitochondrial Alterations with Aging and IPF

**Figure 1:**
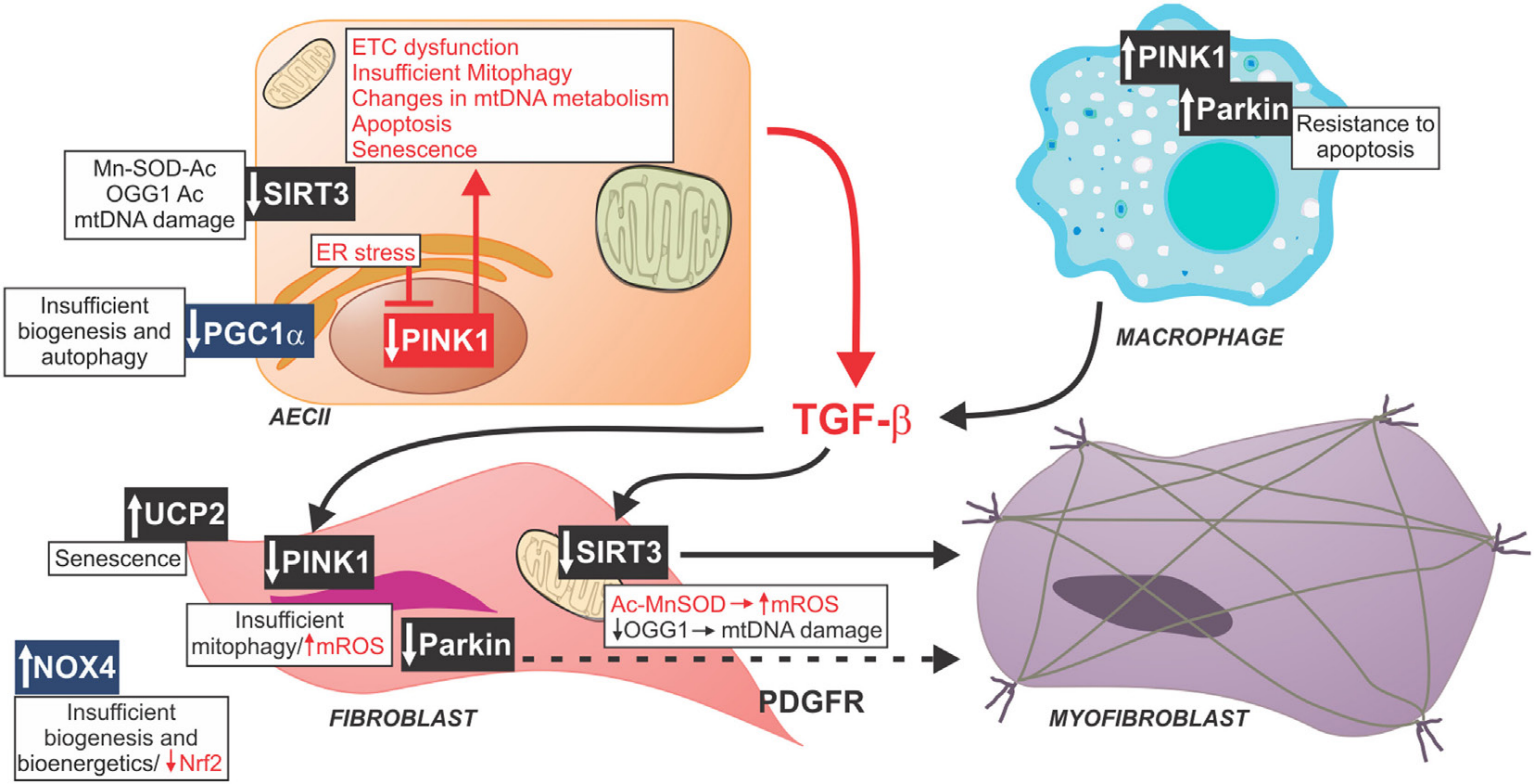
Schematic of profibrotic pathways mediated by mitochondrial dysfunction. Reduced PGC1α in fibrotic lungs has been associated with insufficient biogenesis and diminished autophagy. Similarly, decreased PINK1, a key modulator of mitophagy, and decreased SIRT3 have been found in AECII from fibrotic lungs associated with mitochondrial dysfunction, and increased activation of TGF-β. Profibrotic macrophages exhibit increased rates of mitophagy and resistance to apoptosis resulting in greater release of TGF-β. TGF-β affects mitochondrial function in fibroblasts through decreasing PINK1, Parkin, and SIRT3. Idiopathic pulmonary fibrosis fibroblasts have increased mtDNA damage, mitochondrial dysfunction, impaired mitochondrial biogenesis, and increased rate of senescence. All of these factors contribute to in fibroblast-to-myofibroblast differentiation. Abbreviations: Ac, acetyl group; ETC, type II alveolar epithelial cell electron transport chain, Mn-SOD, manganese super oxide dismutase; mtDNA, mitochondrial DNA, mtROS, mitochondrial reactive oxygen species; NOX4, NADPH oxidase 4; Nrf2, nuclear factor (erythroid-derived 2)-like-2 factor; OGG1, 8-oxoguanine DNA glycosylase 1; PDGFR, platelet-derived growth factor receptor; PINK1, PTEN-induced putative kinase 1; UCP2, uncoupling protein 2; PGC-1α, peroxisome proliferator-activated receptor gamma coactivator 1-alpha.

Over the past few decades, our understanding of the function and role of mitochondria has moved beyond the initial view of mitochondria as nothing more than a cellular energy generator producing ATP through oxidative phosphorylation (OXPHOS). Mitochondria are now understood to have their own life cycle consisting of biogenesis, fission/fusion dynamics, and recycling through macroautophagy (mitophagy), and to act as an important bidirectional signaling platform, communicating with the nucleus as well as other organelles. Multiple signaling pathways converge and interact to regulate the linked processes of mitochondrial energetics, biogenesis, production of reactive oxygen species (ROS), mitochondrial DNA (mtDNA) preservation and repair, and mitophagy. Dysregulation of many of these regulatory mechanisms that control mitochondrial function have recently been identified in the epithelial cells, fibroblasts, and macrophages in IPF lungs (3). Mitochondrial dysfunction in IPF lung cells contributes to maladaptation to cellular stress, creating vulnerability to injury and promoting the development of pulmonary fibrosis (3–6) (Figure 1).

## Mitochondrial Biogenesis

Mitochondrial biogenesis, the process of producing additional mitochondria and by extension cellular energy production capacity, is under the control of master regulators PPARγ coactivator-1α (PGC-1α) and PGC-1β, which are nutritional sensors able to induce expression of nuclear respiration factors 1 and 2 (NRF). NRFs upregulate expression of the nuclear and mitochondrial mechanisms necessary for mitochondrial biogenesis (7). With aging, the capacity for mitochondrial biogenesis declines through age-related reduction in upstream activators of PGC-1α and PGC-1β such as AMP-activated protein kinase (AMPK) as well as p53-mediated, senescence-associated repression of these key regulators of mitochondrial biogenesis (8, 9). Moderate exercise, calorie restriction, and resveratrol supplementation have been shown to increase PGC-1α activity and ameliorates aging-related decline in mitochondrial biogenesis (10–12). Expression of PGC1a is reduced in lungs from IPF patients and in fibrotic mouse lungs after bleomycin treatment. Highlighting the critical role of mitochondrial biogenesis in the susceptibility to lung fibrosis, mice deficient in PGC1a are more susceptible to bleomycin-induced lung fibrosis. Thyroid hormone (T4) signaling is known to restore mitochondrial health and function through a PGC-1α-dependent pathway (13, 14). Concordantly, experimental provision of aerosolized T3 and a T4 mimetic attenuated bleomycin- and TGF-β-induced fibrosis in mouse models. T3 supplementation was also associated with reduced alveolar epithelial cell apoptosis, improvement in mitochondrial electron transport chain function, and normalization of swollen mitochondrial morphology. This improvement was not seen in PGC-1α knockout mice. These investigations were conducted in response to the finding of increased level and activity of iodothyronine deiodinase 2 in IPF lungs, which is the enzyme responsible for conversion of inactive T4 to the physiologically active T3 form. This is presumed to be an adaptive response to increase the availability of active T3 in fibrotic IPF lung (15).

Idiopathic pulmonary fibrosis and experimental models of pulmonary fibrosis are associated with telomere shortening and DNA damage, which activates the DNA damage sensor poly[ADP-ribose] polymerase 1 (PARP-1) and the checkpoint inhibitor p53. These signaling mechanisms feedback to reduce activation of PPARγ coactivators and reduce mitochondrial biogenesis (9, 16). Impaired mitochondrial biogenesis in IPF potentially creates a mismatch in cellular energy demand and production capacity with resulting mitochondrial dysfunction. Additionally, the ROS-producing enzyme NADPH oxidase-4 (Nox4), which is upregulated in IPF lung fibroblasts, represses mitochondrial biogenesis through direct effect on NRF2 and mitochondrial transcription factor A (TFAM) independent of PGC-1α (17, 18). While these pathways have been demonstrated to play a role in age and disease-related alterations in cellular bioenergetics, and the downstream consequences, such as ROS production, DNA damage, and induction of senescence, are features of lung fibrosis, additional translational investigations in IPF lungs are needed to firmly establish the role of these perturbations in the development of IPF.

## Mitophagy

Mitophagy is a selective and adaptive response that targets mitochondria for turnover, regulates the number of mitochondria to match cellular energy needs, and removes damaged and dysfunctional mitochondria that can cause cellular stress. Mitophagy and mitochondrial turnover are important processes in maintaining cellular integrity and health. Mitochondrial damage, whether from metabolic alterations, increased ROS production, or an accumulation of mtDNA damage and mutations affecting transcription, results in a reduction of mitochondrial transmembrane potential, reduced ATP production, further increases in ROS, and leakage of mitochondrial contents into the cytosol that, if left unchecked, would eventually lead to cellular injury and apoptosis (19–23).

Selective mitophagy of damaged mitochondria occurs through PTEN-induced putative kinase 1 (PINK1)-Parkin signaling with PINK1 acting as a sensor of mitochondrial membrane depolarization and subsequently activating Parkin, which labels the dysfunctional mitochondrion for trafficking to the autophagosome (19, 24). With aging, a decrease in PINK1 has been observed coupled with a decrease in markers of autophagy and an increase in the size of mitochondria (5, 25). These findings demonstrate that capacity for mitophagy declines as we age.

Deficient mitophagy has been associated with IPF and development of pulmonary fibrosis in response to injury (5, 16, 25–27). Deficiency or dysfunction of multiple mediators of mitophagy has been implicated in the pathobiology of IPF making maintenance or augmentation of mitophagy an attractive avenue of potential intervention. Our work has shown that type II alveolar epithelial cells from IPF lungs are deficient in PINK1 resulting in an accumulation of dysmorphic mitochondria with reduced transmembrane potential, reduced electron transport chain (ETC) activity, increased ROS production, and increased opening of the mitochondrial permeability transition pore. PINK1 knockout was also found to be sufficient to recapitulate this mitochondrial phenotype and confer vulnerability to fibrosis in response to lung injury (5). PINK1 expression has been found to diminish with age and persistent endoplasmic reticulum stress. ER stress has been shown to induce expression of ATF3, a transcriptional repressor of PINK1, in alveolar epithelial cells (28). Interestingly, deficiency of PINK1 in lung epithelial cells was also associated with upregulation of markers of senescence (p16 and p21) and increased levels of TGF-β expression, a key mediator of fibrogenic processes in IPF (5, 28).

As adaptive responses, autophagy and mitophagy are essential to maintaining normal fibroblast function and preventing apoptosis. Impairment of mitophagy, mediated by Parkin deficiency, in IPF lung fibroblasts has been associated with increased deposition of extracellular matrix under profibrotic conditions such as exposure to TGF-β. Defects in mitophagy and autophagy result in increased production of ROS and activation of platelet-derived growth factor receptor (PDGFR)/mammalian target of rapamycin (mTOR) signaling pathways that enhance fibroblast to myofibroblast transformation (24). Supporting these data, Parkin-deficient mice develop more severe lung fibrosis. Induction of autophagy and treatment with antioxidants reduced fibroblast to myofibroblast differentiation, myofibroblast proliferation, and fibrogenesis suggesting that this process is driven by increased mitochondrial ROS (24, 25). Indeed, we now recognize that a subset of surfactant protein C mutations, which causes a familial form of pulmonary fibrosis, acts through mistrafficking of surfactant protein C, accumulation within endosomes, and a late block of macroautophagy and mitophagy within type II alveolar epithelial cells. This results in proteostasis and an accumulation of dysfunctional mitochondria with increased mitochondrial mass but decreased transmembrane potential, which may increase susceptibility to pulmonary fibrosis when an additional “second hit” stressor is present (29).

TGF-β has been shown to variably affect key mediators of mitophagy and promote fibrogenesis. Treatment of alveolar epithelial cells with TGF-β results in initial stabilization of PINK1 on the surface of mitochondria and induction of mitophagy, which appears to be an adaptive response to increased ROS production due to TGF-β’s effect on the ETC (30). Longer term exposure to TGF-β results in impaired mitophagy through downregulation of phosphatase and tensin homolog (PTEN), and a subsequent reduction in PINK1 expression (25). Another potential activator of autophagy is T4, which is a well-known activator of AMPK and inhibitor of mTOR (15). Supporting this role of T4 signaling in PINK1-mediated mitochondrial homeostasis and mitophagy, the therapeutic effect of inhaled T3 in mouse models of lung fibrosis required an active PINK1 signaling pathway (15).

On the contrary, pulmonary macrophages isolated from IPF lungs demonstrate increased mitophagy. IPF macrophages are a source of activated TGF-β, and macrophage resistance to apoptosis is necessary for disease progression. IPF macrophages activate TGF-β by producing mitochondrial H_2_O_2_ through activation of the pro-survival kinase, protein kinase B (Akt1). Increased Akt1 activation and ROS production induce mitophagy as a protective measure and prevent macrophage apoptosis, which stabilizes macrophages to release additional TGF-β and promote local fibroblast activation and proliferation. Blocking mitophagy in alveolar macrophages is protective against bleomycin-induced fibrosis (27). This finding exemplifies the role of mitophagy as an adaptive survival response that, in this case, serves to preserve and promote macrophage signaling.

## Mitochondrial OXPHOS

The processes of mitochondrial respiration and OXPHOS are the highly efficient and preferable means of ATP production, but a natural byproduct of these processes is superoxide and hydrogen peroxide production, which is produced in increasing quantities by dysfunctional mitochondria (31). Mitochondrial ROS (mtROS) serve as a signaling mechanism acting at multiple points throughout the cell that serves to inactivate phosphatases, activate select kinases, and facilitate hypoxia-inducible factor 1-α (HIF-1α), p53, and NF-κB signaling pathways (32–36). Interestingly, mtROS levels have a biphasic effect. At low levels, mtROS stimulate an increase in antioxidant capacity, referred to as mitohormesis, which has a protective effect and has been associated with longevity and resistance to cellular injury. In higher concentrations or with sustained production, ROS propagates free radicals that cause oxidative damage to lipids, proteins, and DNA (37).

Control of OXPHOS and energy production occurs through cooperative signaling mechanisms at mitochondrial, cytosolic, and nuclear levels in order to balance OXPHOS, energetic demands of the cell, and nutrient supply. This complex process limits production of ROS. Additionally, mitochondria have multiple mechanisms for reducing mtROS, but effective regulation of ROS production and mitigation declines with aging and potentiates susceptibility to lung injury and fibrosis (18, 38, 39). Aging is associated with lower ATP production and increased ROS (40). Moreover, older animals have been shown to accumulate higher levels of oxidized proteins in response to lung injury suggesting waning cellular antioxidant defense systems (41).

A profibrotic environment promotes mitochondrial dysfunction in pulmonary epithelial cells. Studies in Mv1Lu cells demonstrate that treatment with TGF-β1 downregulates mitochondrial ETC function, particularly at complex IV, resulting in loss of mitochondrial transmembrane potential and increased mtROS production (42). Increased ROS serves to oxidize and activate latent TGF-β1 creating a self-reinforcing cycle with potential to recruit fibroblasts and promote fibrogenesis (30). This correlates with the findings that type II alveolar epithelial cells from IPF lungs have reduced ETC complex I and IV activity (5), and IPF fibroblasts have reduced ATP content and reduced rate of oxygen consumption indicating poor mitochondrial function (43).

While the effectiveness of antioxidant supplementation in preventing diseases of aging such as IPF has been disappointing and, in some cases, detrimental, overexpression of an antioxidant protective mechanism in mitochondria has shown effectiveness in abrogating the fibrotic response to bleomycin-induced lung injury. Mice that overexpress mitochondria-targeted human catalase predictably show lower levels of mtROS, but also display reduced mtDNA damage and fragmentation with decreased pulmonary fibrosis in response to oxidative injury from asbestos or bleomycin (44). This raises the possibility that mitochondrial-targeted antioxidants may prevent or abrogate the development of IPF by scavenging mtROS at the source.

## mtDNA Damage

Mitochondrial DNA is significantly more susceptible to oxidative injury than nuclear DNA, likely due to proximity to the source of ROS, lack of protective histones, and relative paucity of mtDNA repair mechanisms (45). Indeed, oxidative DNA damage is a key mechanism of injury in the commonly used model of bleomycin-induced pulmonary fibrosis (46). In comparison with the nucleus, mitochondria contain fewer and less efficient mechanisms for correcting mutations and repairing DNA damage. Mitochondrial transcription factor A (TFAM) is a key regulator of mtDNA transcription and replication, which also functions to induce *U*-shaped bends in mtDNA. These protective nucleoids can act to sequester damaged DNA and prevent transcription until base excision repair enzymes can correct the damage (47). The base excision repair enzyme, 8-oxoguanine DNA glycosylase 1 (OGG1), plays a critical role in repair of mtDNA oxidative damage, OGG1 deficiency or inactivation predisposes to pulmonary fibrosis (26, 48). The processes of mtROS production and mtDNA damage are intricately linked, and oxidative injury and cellular stress drive apoptotic and senescence pathways that may contribute, at least in part, to both aging and to lung injury and fibrosis. Additional studies in IPF lung-derived cells or tissues are needed to establish the role of mtDNA damage in IPF.

Owing to the evolutionary origin of mitochondria as bacterial endosymbionts, mtDNA contains CpG-rich sequences, which have the potential to act as damage-associated molecular patterns and activate innate immune system mechanisms when they escape the mitochondrial milieu. This pathway may contribute to a pro-fibrotic microenvironment (49). Recently, it has been shown that IPF fibroblasts undergo glycolytic reprogramming associated with release of mtDNA into the cellular interstitium and ultimately into circulation. Furthermore, increased plasma levels of mtDNA were associated with an increase in all-cause mortality suggesting potential use of circulating mtDNA as a biomarker for progression or severity of IPF (50).

## Sirtuins are Key Mediators of Mitochondrial Health Implicated in IPF

Deficiency of the mitochondrial NAD-dependent deacetylase, SIRT3, has been shown to confer susceptibility to epithelial cell apoptosis and fibrosis in both bleomycin and asbestos models of lung injury (48). SIRT3 is an active enzyme in ROS detoxification as well as mtDNA protection and repair pathways, and its dependence on NAD+ as a cofactor directly links SIRT3’s function to cellular bioenergetics and TCA cycle activity (51). In the mitochondrion, SIRT3 is implicated in regulation of two redox mechanisms. SIRT3 deacetylates and activates manganese superoxide dismutase (MnSOD) and isocitrate dehydrogenase 2 (IDH2) (52, 53). Low levels of SIRT3 result in increased acetylation and decreased MnSOD activity and an accumulation of mtROS and mtDNA oxidative damage (53). The matrix protein IDH2 is a key provider of the reducing agent, NADPH, necessary for proper functioning of protective antioxidant pathways (52). The mtDNA repair function of OGG1 is also regulated by SIRT3. In the absence of SIRT3, OGG1 is increasingly acetylated and deactivated resulting in unchecked mtDNA damage and lung epithelial cell apoptosis (44, 48).

SIRT3 knockout mice have also been shown to develop increased expression of TGF-β and fibrosis in multiple organs as they age. This has been shown to occur through increased acetylation and deactivation of glycogen synthase kinase-β and stabilization of profibrotic transcription factors, such as smad3 and β-catenin, independent of SIRT3’s effect on MnSOD activity (54). Multiple SIRT3-dependent pathways play important roles in preventing mitochondrial damage and vulnerability to fibrosis.

Another sirtuin, SIRT1, has been established to play a role in preventing both aging-related functional decline and age-related diseases such as IPF (55). SIRT1 is a nuclear NAD-dependent deacetylase, but one of its key functions is in modulating mitochondrial biogenesis and function through nuclear-to-mitochondrial signaling pathways (56). These pathways have demonstrated how nuclear DNA damage and telomere shortening can drive mitochondrial dysfunction. Telomerase reverse transcriptase deficiency and shortened telomeres are strongly linked with the development of pulmonary fibrosis, and this chromosomal shortening activates the DNA damage sensor and checkpoint inhibitor p53. Activated p53 represses expression of a key mediator of mitochondrial biogenesis, PPARγ coactivator-1α (PGC-1α) resulting in inefficient OXPHOS and increased ROS production (57). SIRT1 deacetylates and inactivates p53 restoring normal mitochondrial function (9, 56). The nuclear DNA damage sensor, poly[ADP-ribose] polymerase 1 (PARP-1), consumes NAD+ when activated and decreases activity of SIRT1 and PGC-1α. SIRT1 and PGC-1α have also been shown to translocate to the mitochondria and may assist in stabilization of mtDNA (16). Fasting and caloric restriction, long of interest in increasing longevity, and reducing functional decline with aging, increase SIRT1 protein levels and activity (51). SIRT1 translation is inhibited by the microRNA miR-34a, which is highly expressed in models of lung injury. SIRT1 deficiency results in increased acetylation of p53, which acts to induce expression of miR-34a, creating a self-reinforcing cycle and predisposing to development of fibrosis (58). In a bleomycin model of lung injury, upregulation of SIRT1 attenuated fibroblast activation and the development of fibrosis (59). Sirtuins clearly have complex functions in modulating mitochondrial biogenesis, function, ROS production, and mtDNA protection, and this area of investigation has potential to shed new light on the pathogenesis of IPF and other diseases of aging.

## Metabolic Reprogramming in IPF

**Figure 2:**
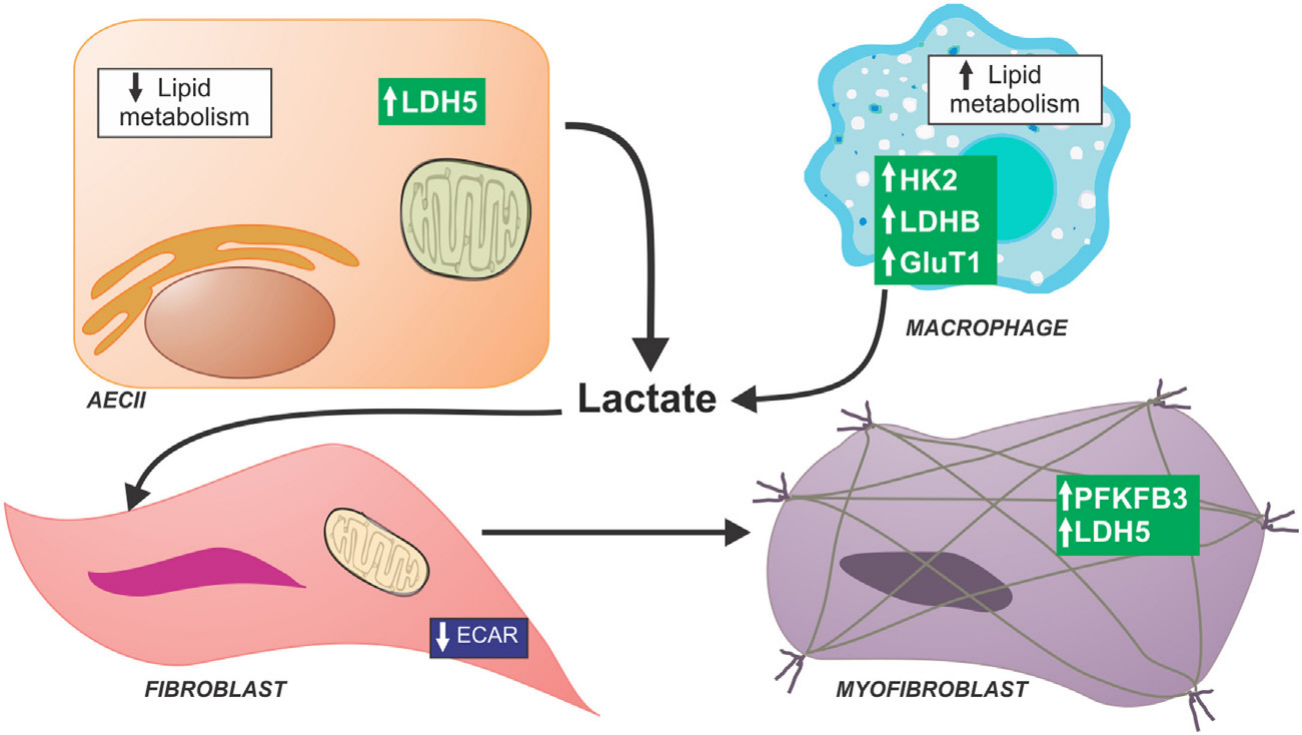
Schematic of metabolic dysfunction in lung fibrosis. Idiopathic pulmonary fibrosis (IPF) lung have a generalized decrease in late stages of glycolysis compared with normal lungs; however, increased levels of lactate were found in IPF lungs suggesting that all products of glycolysis are shuttled toward lactate production. Several studies have been focused in metabolic alterations of specific cell compartments in the IPF lung. IPF lung fibroblasts show decreased glycolytic function determined by extracellular acid production. IPF myofibroblasts have been reported to have higher expression of glycolytic enzymes and lactate content. Single cell RNA sequence data in alveolar epithelial cells show low expression of enzymes necessary for lipid metabolism. By contrast, alveolar macrophages isolated from bleomycin-treated mice show glycolytic reprogramming and increase fatty acid oxidation. Abbreviations: AECII, type II alveolar epithelial cell; GluT1, glucose transporter 1; HK2, hexokinase 2; LDH5, lactate dehydrogenase 5; LDHB, lactate dehydrogenase B; PFKFB3, 6-phosphofructo-2-kinase/fructose-2,6-bisphosphatase 3.

Fibrotic lung tissue in IPF has been shown to have increased metabolic activity as demonstrated by increased FDG uptake on PET scan, and increased PET activity correlates with disease progression and mortality (60). Cell-type specific metabolic changes occur in the IPF lung (Figure 2). Type II alveolar epithelial cells have downregulation of genes involved in lipid synthesis and metabolism identified through single cell RNA sequencing (61). Fibroblasts have been shown to undergo a metabolic shift away from the highly efficient method of ATP production, OXPHOS, to the less efficient method of glycolysis despite adequate oxygen to continue OxPhos (62–67). Additionally, alveolar macrophages in fibrotic lung tissue increase fatty acid oxidation in response to the shift to glycolysis (66). A similar metabolic shift was initially observed in cancer cells and was eponymously dubbed the Warburg effect after its discoverer (68). The Warburg-like metabolic reprogramming observed in IPF cells results in increased glucose uptake and an accumulation of TCA cycle metabolites and byproducts that act as signaling mechanisms. Kottmann and colleagues demonstrated that glycolytic flux increases lactate production and lowers the local tissue pH resulting in increased activation of TGF-β, stabilization of the transcription factor HIF-1α, and increased transcription of lactate dehydrogenase 5 (LDH5), which synergizes with TGF-β to induce differentiation of fibroblasts to myofibroblasts (62). LDH5 produces lactate, which can be exported by monocarboxylate transporter (MCT)-4 and may be taken up by adjacent cells expressing MCT-1. Shuttled lactate can be oxidized to pyruvate providing additional energy through the TCA cycle and driving OXPHOS. This phenomenon is termed the reverse Warburg effect and promotes fibroblast proliferation and increased levels of mtROS.

Primary myofibroblasts derived from IPF lungs and lung fibroblasts treated with TGF-β demonstrate increased lactate contents. Additionally, IPF myofibroblasts showed increase in expression of glycolytic enzymes including 6-phosphofructo-2-kinase/fructose-2,6-biphosphatase 3 (PFKB3), an enzyme that increases levels of fructose-2,6-bisphosphate and drives glycolysis (63). Glycolytic reprogramming in fibroblasts is, at least partially, driven by TGF-β. Treating normal fibroblasts with TGF-β has been shown to increase transcription of phosphofructokinase (PFK) and hexokinase 2, two key rate-limiting glycolytic enzymes (63). Increased lactate production may be due to shunting of glycolytic products toward anaerobic metabolism due to concurrent mitochondrial dysfunction.

By contrast, a recent study comparing IPF lung tissue to normal human lung using a combined metabolomic and microarray analysis of key metabolic enzymes showed a decrease in the late-stage glycolytic products, fructose 1,6-bisphosphate and phosphoenolpyruvate, and a decrease in PFK and PFKFB3, suggesting an overall reduction in glycolysis in IPF whole lung tissue (69). These findings are in concordance with recent observations in fibroblasts derived from IPF lungs. IPF fibroblasts trended toward having a lower rate of glycolysis, as measured by extracellular acidification rate, and had lesser glycolytic flux in response to TGF-β stimulation compared to normal lung fibroblasts (43). The latter finding may represent a diminished capacity to respond to TGF-β after chronic exposure to this profibrotic cytokine *in vivo*.

Despite these differences, it is known that TGF-β can mediate metabolic reprogramming. TGF-β induces expression of the facultative glucose transporter, glucose transporter 1 (GLUT1), *via* canonical Smad and PDGFR signaling resulting in increased cellular glucose uptake. Inhibiting GLUT1 upregulation is protective against bleomycin-induced fibrosis (64), and GLUT1 activation of AMPK is integral to fibroblast activation (67). Since metabolic reprogramming appears to be a critical step in promoting fibroblast-to-myofibroblast differentiation, preventing this shift is an intriguing target in attenuating IPF. However, further studies are needed to confirm the metabolic changes in different cell compartments in the IPF lung and determine how these alterations might modify disease progression.

## Mitochondrial Dysfunction and Senescence

Inducing senescence and apoptosis in fibroblasts are important mechanisms in the wound healing response after injury (70); on the contrary, the aberrant deposition of extracellular matrix seen in IPF is associated with an accumulation of apoptosis-resistant senescent cells (39, 43, 71, 72). Mitochondrial dysfunction has been shown to induce cellular senescence, a process termed mitochondrial dysfunction-associated senescence. In addition, mitophagy defects seem to contribute to a cellular senescence phenotype, which we have observed in the setting of mitochondrial dysfunction induced by PINK1 deficiency *in vivo* (28). Dysfunctional mitochondria are less efficient at oxidizing NADH to NAD+ resulting in a reduced NAD+/NADH ratio, activation of AMPK and p53 resulting in a senescent phenotype (73). Senescent IPF lung epithelial cells, fibroblasts, and myofibroblasts secrete a cell type-specific profile of pro-fibrotic and pro-inflammatory cytokines known as the senescence-associated secretory phenotype (SASP) (72, 74). Senescent fibroblasts exhibit an increased level of the ROS-generating enzyme Nox4 and low levels of the key mediator of cellular antioxidant response pathways, NFE2-related factor 2, and this redox imbalance plays a role in maintaining the senescent phenotype. Treatment with a Nox inhibitor restores the redox balance and results in a decrease in senescent cells and a reduction in fibrosis (18). *In vitro*, mouse model, and *ex vivo* studies in human IPF lung cells have all shown improvement in fibrosis with treatment with a combination of antioxidants (quercetin) and senolytic agents (dasatinib) (72, 74). This approach shows promise as a therapeutic option.

## Future Directions: Targeting Mitochondria as an Antifibrotic Therapy

Research has extensively shown that both aging and IPF are associated with alterations in cellular bioenergetics and mitochondrial homeostasis. Many of these mitochondrial pathways, especially those involved in maintenance of redox balance, mtDNA protection, and reversal of senescence, have shown promise as therapeutic targets in attenuating or ameliorating fibrosis *in vitro* and in animal models of pulmonary fibrosis. These early investigations of the role of altered bioenergetics in the development of fibrotic lung disease pave the way for translational research to implicate these pathways in human IPF. Other pathways, specifically those involved in maintaining normal mitophagy and turnover of dysfunctional mitochondria and mitochondrial-targeted antioxidants, present potential avenues for altering the natural history of pulmonary fibrosis but require further investigation to identify a point of intervention. Few potential interventions appear ready for human trials to restore mitochondrial biogenesis and mitophagy in alveolar epithelial cells and use of combination senolytic and antioxidant agents to remove senescent cells from injured lung and prevent propagation of SASP signaling (75). These approaches benefit from a history of therapeutic use and safety in treatment of human disease.

There is considerably more to learn about the role of mitochondrial dysfunction in the development and maintenance of the pro-fibrotic conditions that drive IPF, regarding the interplay between mitochondrial and nuclear signaling in overall cellular dysfunction, telomere shortening, and mtDNA damage, and the varying effects of mitochondrial dysfunction in the different cell types (epithelial cells, macrophages, fibroblasts, and myofibroblasts) involved in the development of IPF. Additionally, cellular perturbations seen with normal aging such as decreased macroautophagy and cellular proteostasis have the potential to incite mitochondrial dysfunction and changes in cellular bioenergetics. Development of animal models capable of isolating defects in mitochondrial homeostasis to a particular cell type may be revelatory of the role each cell type plays and shed light on the optimal targets for intervention. Particular attention to the effects of mitochondrial dysfunction on telomere length in the resident progenitor cells of the lungs responsible for regenerating the alveolar epithelium may present novel approaches for preventing stem cell exhaustion in IPF.

While many of these pathways have shown potential as therapeutic targets in isolation, an integrated approach to the bioenergetic and homeostatic changes that are seen with aging and IPF, such as telomere shortening, reduced mitochondrial biogenesis, increased ROS production, and increased nuclear and mtDNA damage, is essential to determining the pathways and therapeutic targets that are most likely to affect the natural history of IPF.

## Author Contributions

DZ wrote the manuscript along with MB. The manuscript was conceptualized, supervised and modified by ALM and MR. All authors offered intellectual contribution and approved the manuscript.

## Conflict of Interest Statement

The authors declare that the research was conducted in the absence of any commercial or financial relationships that could be construed as a potential conflict of interest.
